# The effects of preventive aerobics mix on body composition in healthy adult women

**DOI:** 10.3389/fphys.2023.1132619

**Published:** 2023-03-03

**Authors:** Omer Špirtović, Ilma Čaprić, Mima Stanković, Dušan Đorđević, Benin Murić, Izet Kahrović, Rifat Mujanović, Raid Mekić, Borko Katanić, Igor Jelaska, Goran Sporiš

**Affiliations:** ^1^ Department of Biomedical Sciences, State University of Novi Pazar, Novi Pazar, Serbia; ^2^ Faculty of Sport and Physical Education, University of Nis, Nis, Serbia; ^3^ Montenegrin Sports Academy, Podgorica, Montenegro; ^4^ Faculty of Kinesiology, University of Split, Split, Croatia; ^5^ Faculty of Kinesiology, University of Zagreb, Zagreb, Croatia

**Keywords:** fitness, recreation, aerobics, women, body composition

## Abstract

The aim of this research was to determine the influence of a 12-week program of aerobics mix on the parameters of body composition in healthy adult women. The research has been performed in a sample of 64 women participants, and it is divided into two groups, an experimental group (E), made of 34 women participants (age 32 ± 1.8 years), and a control one (C), made of 30 women participants (age 33 ± 0.5 years). Their anthropometric and body composition were evaluated using the following respective parameters: body height, body weight, body fat percentage, muscle mass percentage, skinfold–back (KNL), skinfold–upper arm (KNN), skinfold–stomach (KNT), and skinfold–thigh (KNNK). After mix aerobics realization, among the women of the E group, there is a reduction of body mass by −2.5% and waist size by −3.39%, while muscle mass increased by 2.26%. With all skinfolds within the participants of the E group, there is a statistically important gained reduction of *p* < 0.05 at the final measuring, compared with the initial one (upper arm −21.10%, stomach −14.36%, back −20.58%, and upper leg −13.98%). The reduction of body mass percentage was −10.59%, and that of body mass index was −2.5%. Based on the gained results, it can be concluded that the mix program was efficient in the reduction of the subcutaneous fat tissue and visceral fat and also influential on the increase of muscle mass.

## 1 Introduction

According to many research studies, dosed physical activity is crucial for human health and the development of one’s abilities and characteristics (Galloway and Jokl, 2000; Hu et al., 2001; Obidovna and Sulaymonovich, 2022). It is known that a human being, influenced by the scientific–technological revolution and a higher level of mechanization and automatization, is prone to having a lower level of physical activity (Blair et al., 2004). The result of such a lifestyle can impair human health when looking through the increased mass contents and a higher concentration of body mass compared to non-fat body mass (Milanović et al., 2011). Body composition influences the manifestation of certain physical abilities to some degree and, thus, represents an important indirect indicator of health fitness level, which is spontaneously changed upon exercise.

The changes in body composition depend on numerous interrelated factors, such as body mass, gender, age, genetic factor, type of training activity, and diet regime (Stojiljković et al., 2005). The increase in body mass and a high level of obesity represent one of the most serious factors of health risk (Saris et al., 2003). Programed exercises with a regular diet regime have a significant influence on body composition (Paoli et al., 2017). Fitness activities, such as programed body exercising, where each individual, according to the individual potential, can choose the activity type, time, and place, under the expert control of a trainer and a doctor, are the very measure for man’s health (Špirtović et al., 2021). An active lifestyle accomplishes positive changes in muscle functioning. When the muscle system works, fat burns easily and more efficiently (Wargo and Kurath, 2012), and for these reasons, it is necessary to choose an adequate recreational program that can proficiently contribute to obesity reduction, if not its elimination, and act preventively against locomotor apparatus disease and the cardiovascular and nerve system (Aćimović and Špirtović, 2012; Wargo and Kurath, 2012). Numerous studies have confirmed a positive effect of different aerobic programs on the changes in body composition and anthropometric characteristics of a man (Osei-Tutu and Campagna, 2005; Rahimi, 2006). Aerobic training has a massive influence on body composition changes, as it causes body weight and body fat reduction when an adequate diet is followed (Jorgić et al., 2011; Milanovic et al., 2012). In addition to walking and running, aerobic training includes different music models such as steps, turns, hops, jumps, and other moves that help reduce body weight and change body composition (Milanovic et al., 2012). One of the models of aerobic exercises containing such and similar activities is the mix aerobics exercising program. Although there are studies that indicate that aerobic training in women affects body composition, most of those studies examined the combined effect of aerobic exercise and nutrition (Christiansen et al., 2009; Ross et al., 2020). Mostly, the treatments were carried out on obese women (Foster‐Schubert et al., 2012; Cheng et al., 2018). However, it should be pointed out that there has been no study that examined the impact of aerobic training on the body composition of women in Serbia. Based on that, this study examines the impact of exclusive aerobic training on the general population of women in Serbia. In this regard, the aim of this study was to determine whether and to what extent a 12-week preventive program of mix aerobics influences body composition changes in healthy adult women.

## 2 Materials and methods

### 2.1 Participants

A total of 64 out of 68 women, who were randomly divided into two groups, finished the training program, and participated in the initial and final measuring. Three participants dropped out of the E group due to a lack of exercising motivation and unavailability to participate in the research, and one participant in the C group was sick during the final testing. The E group consisted of 34 women (age 32 ± 1.8 years), while the C group consisted of 30 women (age 33 ± 0.5 years). The participants of the E group took part in the exercising program of mix aerobics, conducted at the State University of Novi Pazar facilities. They did not have any chronic diseases or any obstacles in the work of their locomotor system; these could have limited their scope of movement during training. During this experiment, they did not participate in other organized forms of physical activities, but they performed their everyday activities and followed their regular nutrition. The participants of the C group did not participate in any form of organized physical activities, except their everyday activities, and they were advised to continue with their everyday activities during the period of the experiment. All participants gave written consent to participate in the study. This study was approved by the Ethics Committee of the University of Novi Pazar (Ref. No. 110-01/23) and was conducted under the Declaration of Helsinki.

### 2.2 Variables sample

The effects of the aerobics program of exercise, mix aerobics, on body composition were studied based on the effect of the training on skinfold thickness, body fat, and body mass percentage. The following measures were used: body fat percentage, muscle mass percentage, body height, body weight, skinfolds–subscapular, skinfold–triceps, skinfold–abdomen, and skinfold–thigh. Anthropometric measurement was conducted by using standard measuring instruments as prescribed by the International Biological Program (IBP). Skinfolds were measured by using a Harpenden Skinfold Caliper (Rutherford et al., 2011; Naz et al., 2017). Body weight, body fat percentage (BF%), and muscle mass percentage (MM%) were calculated using an apparatus for bioelectrical impedance, TANITA UM-72 (monitor of body composition, Tanita Corp, Tokyo, Japan) (Vasold et al., 2019), which has been used in many studies (Mustedanagić et al., 2016; Bratic et al., 2022). For measuring body height, anthropometry per Martin was used, and the result was read to within 0.1 cm error. For the calculation of BMI values, we used a standard procedure based on the formula BMI = body weight (kg)/body height (m^2^). After the initial measuring, a 12-week exercising program was conducted, followed by final measuring, in order to determine the accomplished training effects. During the testing, the air temperature in the facility ranged from 23°C to 26°C, and both measurements always started at 09:00 a.m. and finished by 12:00 a.m., to reduce daily variations in measuring.

### 2.3 Exercising program

The aim of the experimental model of mix aerobics (Table 1) and of aerobics training with music is to determine its influence on the body composition of the E group participants. During its realization, the participants voluntarily approached for measuring, and all pedagogical and physiological principles were respected during the process of exercising. A total of 36 training sessions took place during the 12 weeks, and each training was realized with a different speed and appropriate music accompanying it. Exercises were performed in the introductory part of the training, and their aim was to warm up and prepare the locomotor apparatus for higher burden, increase organism functions to a higher level, increase concentration on work and acceptance of music rhythm, and raise heart frequency to 114–125 heartbeats per minute (Brick, 1996). The main training part lasted for 40 min, and it consisted of aerobics and strength exercises. The aerobic part consisted of 55% of the entire training, and during this part, the participants’ pulse was from 115 to 152 heartbeats per minute. The optimal intensity is calculated based on the maximal heartbeat frequency (220 minus years of age). Each participant independently followed their heartbeat frequency with the assistance of a fitness watch, which had measuring pulse sensors installed. For monitoring heart frequency during physical activities, a Polar V800 was used, and it proved to be a valid and reliable measuring instrument (Caminal et al., 2018). For the second half of the main part of the mix aerobics training model, lasting for 25% of the entire duration, without any passive breaks, an exercising series for strengthening the stomach wall, back muscles, arms and shoulder muscles, abductor muscles, and abductor and gluteal areas was applied. The final training lasted for 5% to 10% of the entire training duration, and it consisted of relaxation exercises and had the aim to calm down the participant, relax musculature, and return vital functions to the normal levels of an individual. In the first month, the training had a smaller burden level and simpler exercises, and then, to accomplish the effect of exercising, the burden was increased gradually and more complex exercises were applied.

**TABLE 1:** Training structure.

Training structure	Duration (60 min)	Activity/contents (%)
Introductory	10%	Walking, running, and hops with arm coordination according to the music rhythm
Main/aerobics	55%	Stepping and wide stepping in front of the stepper; stepping and wide stepping on the stepper: alternating “Tap” step in the middle and sides of the stepper; “Basic step”; “Alternating basic step”; “Tap up”; “V-step”, “Leg lift alternating”; “Turn step”; “Over the top”; “Repeater”
Main/strength	25%	3 arm exercises (10 × 3)
		2 back exercises (12 × 2)
		3 abdominal exercises (15 × 3)
		2 leg exercises (12 × 2) with the necessary help (weights, medical kit, and elastic bands)
Final	10%	Applied attitudes
		Standing: heels sitting; split in a horizontal bowing; sitting in front and spreading legs; in “hurdle seat”

### 2.4 Data processing methods

The analyses of data are performed by using SPSS 25 (Statistical Package for Social Sciences, v25.0, SPSS Inc., Chicago, IL, United States). Descriptive statistics are calculated for all experimental data. In addition, for all variables, the Kolmogorov–Smirnov test was performed before the analyses, and distribution normality and variance homogeneity were tested by Levene’s test. To determine whether there are statistically significant differences between the initial and final measuring of the E and C groups, a T-test for dependent samples, testing for one group in the initial and final state and a T-test for independent samples, testing differences between the groups and these conditions were used. Statistical importance is accepted for values of *p* < 0.05.

## 3 Results

In Table 2, descriptive statistics for variables included in the study are provided. The results are shown separately for the C and E groups for both measuring stages. The range of variable scores is shown without minimal and maximal values since the values have not been changed for more than one measurement unit of each score. Additionally, the participants of the study were all female, with no outliers in body height (min = 156, max = 177) or body weight scores (min = 43.9; max = 70.2). Hence, the sample can be considered a representative one. Levene’s test showed that the variances of all of the samples are homogeneous; that is, there is no significant difference in the variances of scores of all the variables for the C group in comparison to the E group (F < 2.045; *p* > 0.163 > 0.05 for all variables). The Kolmogorov–Smirnov normality test, with Lilliefors correction, showed that the distribution of the variables does significantly differ from the normal distribution for most of the variables included in the analysis. The significant discrepancy of some variables can even be seen from Table 2, where the values of skewness and kurtosis are extreme. This indicates that the parametric methods of statistical analysis should be double-checked with non-parametric methods.

**TABLE 2:** Descriptive statistics of indicator variables in the initial state.

Variable	Group	Mean		St. dev		Skew		Kurt		Range	
		Initial	Final	Initial	Final	Initial	Final	Initial	Final	Initial	Final
Body mass index (BMI)	Con	20.463	20.169	2.863	2.454	0.244	0.893	−0.354	1.333	9.83	9.26
	Exp	20.120	19.617	1.917	1.840	0.285	0.516	1.044	1.022	7.61	7.22
Body height (cm)	Con	168.875	168.875	5.841	5.841	−0.345	−0.345	−0.464	−0.464	20	20
	Exp	168.125	168.125	6.490	6.490	−0.400	−0.400	−0.700	−0.700	21	21
Body weight (kg)	Con	57.675	56.931	8.569	7.955	0.092	−0.063	−1.214	−0.627	25.4	25.4
	Exp	56.956	55.531	6.889	6.627	−0.438	−0.336	−0.774	−0.954	22.6	21.5
Body fat percentage	Con	21.625	22.031	4.208	4.480	−1.849	−1.599	2.818	2.507	14	17
	Exp	21.775	19.469	4.344	3.973	−1.544	−1.156	2.040	1.041	15.4	14
Muscle mass	Con	43,375	45.469	5,045	4.573	0.913	0.648	−0.080	0.652	17	17
	Exp	43,150	44.125	5,087	5.262	0.898	0.915	0.013	0.273	17	18
Waist size	Con	71.750	72.063	4.837	4.946	−0.270	−0.194	−0.645	−1.003	16	15
	Exp	71.813	69.375	5.049	4.777	−0.450	−0.539	−0.156	−0.324	18	17
Arm	Con	11.313	11.594	6.444	6.301	0.028	0.120	−1.781	−1.676	16.5	17.5
	Exp	11.906	9.394	6.406	5.437	0.084	0.173	−1.734	−1.761	17	14
Stomach	Con	15.812	15.969	6.167	6.320	1.166	1.147	0.576	0.440	20	20
	Exp	15.581	13.344	6.117	5.555	1.226	1.124	0.651	0.327	20	17.5
Back	Con	11.219	11.375	4.246	4.319	0.482	0.383	1.436	1.070	17	17
	Exp	11.450	9.094	4.615	3.917	0.702	0.976	1.677	1.547	18.5	14.5
Thigh	Con	20.188	20.313	6.221	6.258	−0.226	−0.280	−0.117	−0.167	22	22
	Exp	19.400	16.687	6.135	5.385	−0.224	−0.092	−0.135	0.031	21.7	19

As we can see in Table 2, in the initial state, the average scores of all variables are not noticeably different. This has been shown *via* T-test for independent samples (t < 0.361; *p* > 0.721 > 0.05 for all variables) and confirmed *via* the Man–Whitney test (Z > −0.755; *p* > 0.450 > 0.05 for all variables). This homogeneity of the sample is an indicator of a proper sample. Namely, there is no possibility of false-positive results for our treatment (exercise and program); i.e., the variability registered in the final state will represent the effectiveness of the treatment. Furthermore, we examine whether the scores of the variables for the C group significantly differ in the final state in comparison to the initial state. In Table 2, we can see there are certain differences. However, in order to determine whether these differences are significant, we use a T-test for dependent samples (for participants in the C group only). The results of the T-test for paired samples have shown no significant differences in the initial state scores in comparison to the final state scores for the C group, for all of the variables (t < 1.755; *p* > 0.096 > 0.05), except the muscle mass percentage (t = −2.822; *p* = 0.013 < 0.05). The results were confirmed *via* the Wilcoxon test (Z > −1.633; *p* > 0.102 > 0.05) for all variables except the muscle mass, for which we have Z = −2.314; *p* = 0.021 < 0.05. These results indicate that the comparison of the final state scores for the C and E groups will be reliable because the scores for all the variables, except muscle mass, have not changed significantly. Additionally, the percentage of muscle mass for the C group has significantly increased in the final state in comparison to the initial state, which indicates that this difference can only cause rejection of our hypothesis. In other words, the mentioned difference does not contribute to the results we tend to obtain. In order to completely analyze the effectiveness of our treatment, we apply two further analysis methods: a T-test for paired samples to see if the scores in the initial state significantly differ in comparison to the final state for the E group and a T-test for independent samples to determine if the score differences for the participants in the C group differ significantly in comparison to the E group in the final state. With the first method, we prove that the improvement upon implementing our treatment is significant, and with the other method, we prove that without programed physical activity, it is difficult to achieve these results.

For the E group, all of the variable scores are significantly higher (t > 6.589; *p* < 0.001) in the initial state than in the final state, except for the muscle mass percentage, which is lower (t = −7.779; *p* = 0.000); see Table 3. The results are confirmed using the Wilcoxon test (Z < −2.661; *p* < 0.008 < 0.05). Hence, the physical characteristics of the participants in the E group have significantly improved in the final state. This is a clear indicator of the effectiveness of the treatment. By analyzing the listed changes in Table 3, we can see that our treatment has affected significant improvements on the physical characteristics of the E group participants. Finally, we provide the T-test for independent samples’ results for scores in the final state, where we compare the scores of the E group to those in the C group. The normality of distribution is not satisfied, so we check the results with the non-parametric Wilcoxon test. Levene’s test has shown no significant difference in variances of these groups (F < 0.968; *p* > 0.333 > 0.05). The results of the T-test have not shown a significant difference in the scores of all the variables. However, the results are inconclusive due to the *p*-value being close to 0.05. Therefore, we have performed the same hypothesis testing *via* the Wilcoxon test and determined that the E group and C group scores are significantly different in the final state for the body fat percentage (Z = −2.456; *p* = 0.014 < 0.05), back (Z = −2.309; *p* = 0.021 < 0.05), and arm (Z = −1.963; *p* = 0.05). For other variables, no significant difference has been identified (Z > −1.649; *p* > 0.099). Referring to the results in Table 3, a significant improvement in the physical characteristics for the participants in the E group is determined.

**TABLE 3:** Results of the T-test for paired samples: The differences of the final state scores compared to the initial state scores.

Variable (initial–final)	Difference mean	St. dev	95% CI for difference mean		t_15**
			Lower	Upper	
Body mass index	0.502	0.286	0.350	0.654	7.030
Body weight (kg)	1.425	0.843	0.976	1.874	6.758
Body fat percentage	2.306	0.835	1.862	2.751	11.053
Muscle mass	−0.975	0.501	−1.242	−0.708	−7.779
Waist size	2.438	0.750	2.038	2.837	13.000
Arm	2.512	1.525	1.700	3.325	6.589
Stomach	2.238	0.830	1.795	2.680	10.781
Back	2.356	1.157	1.740	2.973	8.146
Thigh	2.712	1.331	2.004	3.422	8.154

**p < 0.001 < 0.05.

Though the results have not shown a significant difference for all scores, the difference is observable, and it indicates their cause is the treatment that the participants are subjected to. Namely, all of the E group average scores are lower for the final state, which was the aim of the treatment (Table 2; Table 4.).

**TABLE 4:** Results of the T-test for independent samples: The differences of the control group and experimental group scores in the final state.

Variable (con.–exp.)	Difference mean	t_30
Body mass index	0.552	0.720
Body weight (kg)	1.400	−0.115
Body fat percentage	2.562	0.541^*^
Muscle mass	1.344	0.771
Waist size	2.688	1.563
Arm	2.200	1.057^*^
Stomach	2.625	1.248
Back	2.281	1.565^*^
Thigh	3.625	1.756

**p < 0.05 based on the Wilcoxon test.

## 4 Discussion

The purpose of this study was to determine the effects of a mix aerobics preventive program on the body construction in women. Each training session lasted 60 min, and the exercising intensity was from 60% to 85% of the maximal heart frequency. The applied exercising 3-month program had positive results on all variables. This is evident in the E group, as there was a reduction of subcutaneous fat tissue and an increase in muscle mass. This was the exact aim of this training process and one of the pre-conditions for the good health condition of a physically active person. Since the participants of the C group did not undertake any physical activities, we did not expect any significant changes in the values of subcutaneous mass tissue and body composition. After realization of the mix aerobics program, within the participants of the E group, there was a reduction of body mass by 2.5% and waist size by −3.39%, while muscle mass increased by 2.26%. The programed training caused a reduction of the skinfolds and body fat percentage, and in this way, it contributed to the change in the body mass of the participants. Within skinfolds, these are the noticed significant changes: upper arm: 21.10%, stomach: −14.36%, leg: 20.58%, and thigh: 13.98%. The reduction of the body mass percentage was 10.59%, and for BMI, it was 2.5%. Aerobic exercises of different types (running, walking, and leaping) can help with the improvement and maintenance of body composition characteristics (Kenney et al., 2021) and have benefits in terms of lowering body fat mass (Zouhal et al., 2020), triceps skinfold, BMI, fat mass, and increasing fat-free mass (FFM) (Silva et al., 2014). The mechanisms that led to a reduction in the body mass were physical exercises, increasing energy consumption, and with it, a reduction in body mass, which was not compensated by increased calorie intake. In the study by Pantelić et al. (Pantelic et al., 2013), exercising improves fat usage, which mostly happens during low- and middle-intensity exercising, and carbohydrates were the main source needed during high intensity exercise. The gained positive effects indicate how programed body activities ensure significant transformational impacts when body compensation is in question. This could be noticed in the works of other authors, where this type of research was their subject of interest and who gained similar results (Obrovac, 2015; Hrgetić et al., 2016). Keeping in mind that body fat and cellulite reduction is achieved by performing adequate exercises that “hit” certain body parts which are endangered the most by body fat, the exercise program was based on strength exercises. These exercises were focused on those body parts which are prone to having subcutaneous fat tissue at a higher level: the hips, waist, stomach region, and upper arm, and this gave a positive result (Špirtović et al., 2021). Based on the gained results in this study, it can be concluded that statistically significant differences are found in all variables for subcutaneous fat tissue and body composition assessment within the participants of the E group between the initial and final measuring. It can be concluded that in the final muscle mass measuring, there are no statistically significant differences, although a visible change was noticed. Thus, it is recommended to use the appropriate strength exercises to gain a better effect in an aerobic program. Since the mix program gave significant results regarding the reduction of body mass and skinfolds, it can be recommended as organized exercises in fitness centers for people who want to regulate their body mass and body composition through fitness activities, to remove the excessive subcutaneous fat tissue that also represents one of the conditions for healthier life. Aerobic exercises of a different intensity, mainly aerobic endurance, have benefits in terms of lowering body fat mass (Zouhal et al., 2020), triceps skinfold, BMI, fat mass, and increasing fat-free mass (FFM) (Silva et al., 2014). The main limitations of this study are related to the small sample size and having no control over the calorie intake, so a suggestion for further research would be to carry out the treatment on a larger sample, as well as to compare different aerobic programs to determine which aerobic program gives the best results.

## Data Availability

The raw data supporting the conclusions of this article will be made available by the authors, without undue reservation.

## References

[B1] Aćimović D. Špirtović O. (2012). Teorija i metodika rekreacije. Beograd. [in Serbian].

[B2] Blair S. N. LaMonte M. J. Nichaman M. Z. (2004). The evolution of physical activity recommendations: How much is enough? Am. J. Clin. Nutr. 79 (5), 913S–920S. 10.1093/ajcn/79.5.913S 15113739

[B3] Bratic M. Dosic A. Zivkovic D. Zivkovic M. Bjelakovic L. Stojanovic N. (2022). The effects of the aerobic endurance running program on the morphological characteristics of adolescent girls with different nutritional status. Int. J. Morphol. 40 (5), 1335–1343. 10.4067/s0717-95022022000501335

[B4] Brick L. (1996). Fitness aerobics. Human Kinetics Publishers.

[B5] Caminal P. Sola F. Gomis P. Guasch E. Perera A. Soriano N. (2018). Validity of the Polar V800 monitor for measuring heart rate variability in mountain running route conditions. Eur. J. Appl. Physiol. 118, 669–677. 10.1007/s00421-018-3808-0 29356949

[B6] Cheng C-C. Hsu C-Y. Liu J-F. (2018). Effects of dietary and exercise intervention on weight loss and body composition in obese postmenopausal women: A systematic review and meta-analysis. Menopause 25 (7), 772–782. 10.1097/GME.0000000000001085 29533366

[B7] Christiansen T. Paulsen S. K. Bruun J. M. Overgaard K. Ringgaard S. Pedersen S. B. (2009). Comparable reduction of the visceral adipose tissue depot after a diet-induced weight loss with or without aerobic exercise in obese subjects: A 12-week randomized intervention study. Eur. J. Endocrinol. 160 (5), 759–767. 10.1530/EJE-08-1009 19211707

[B8] Foster‐Schubert K. E. Alfano C. M. Duggan C. R. Xiao L. Campbell K. L. Kong A. (2012). Effect of diet and exercise, alone or combined, on weight and body composition in overweight‐to‐obese postmenopausal women. Obesity 20 (8), 1628–1638. 10.1038/oby.2011.76 21494229 PMC3406229

[B9] Galloway M. T. Jokl P. (2000). Aging successfully: The importance of physical activity in maintaining health and function. JAAOS-Journal Am. Acad. Orthop. Surg. 8 (1), 37–44. 10.5435/00124635-200001000-00004 10666651

[B10] Hrgetić M. Dadić M. Milanović M. Skoblar J. (2016). Utjecaj tromjesečnog fitness programa vježbanja na antropološki status žena srednje životne dobi. Zb. Rad. Zagreb Hrvat. Kineziol. Savez., 354–357.

[B11] Hu F. B. Stampfer M. J. Solomon C. Liu S. Colditz G. A. Speizer F. E. (2001). Physical activity and risk for cardiovascular events in diabetic women. Ann. Intern Med. 134 (2), 96–105. 10.7326/0003-4819-134-2-200101160-00009 11177312

[B12] Jorgić B. Pantelić S. Milanović Z. Kostić R. (2011). The effects of physical exercise on the body composition of the elderly: A systematic review. Facta Univ. Phys. Educ. Sport 9 (4), 439–453.

[B13] Kenney W. L. Wilmore J. H. Costill D. L. (2021). Physiology of sport and exercise. Human kinetics.

[B14] Milanovic Z. Pantelic S. Trajkovic N. Sporis G. Aleksandrovic M. (2012). The effects of physical exercise on reducing body weight and body composition of obese middle aged people. A systematic review. HealthMED 6 (6).

[B15] Milanović Z. Pantelić S. Trajković N. Sporiš G. (2011). Basic anthropometric and body composition characteristics in an elderly population: A systematic review. Facta Univ. Phys. Educ. Sport 9 (2), 173–182.

[B16] Mustedanagić J. Bratić M. Milanović Z. Pantelić S. D. (2016). The effect of aerobic exercise program on the cardiorespiratory fitness and body composition of female college students. Facta Univ. Ser. Phys. Educ. Sport, 145–158.

[B17] Naz H. Mushtaq K. Butt B. A. Khawaja K. I. (2017). Estimation of body fat in Pakistani adult: A comparison of equations based upon skinfold thickness measurements. Pak. J. Med. Sci. 33 (3), 635–639. 10.12669/pjms.333.12806 28811785 PMC5510117

[B18] Obidovna D. Z. Sulaymonovich D. S. (2022). Physical activity and its impact on human health and longevity. Достижения науки и образования. 2 (82), 120–126.

[B19] Obrovac S. (2015). Analiza promjena u sastavu tijela i pojedinim motoričkim sposobnostima pod utjecajem šest tjednog programa vježbanja. University of Zagreb. Faculty of Kinesiology. Department of Kinesiology of.

[B20] Osei-Tutu K. B. Campagna P. D. (2005). The effects of short-vs. long-bout exercise on mood, VO2max., and percent body fat. Prev. Med. Balt. 40 (1), 92–98. 10.1016/j.ypmed.2004.05.005 15530585

[B21] Pantelic S. Milanovic Z. Sporis G. Stojanovic-Tosic J. (2013). Effects of a twelve-week aerobic dance exercises on body compositions parameters in young women. Int. J. Morphol. 31 (4), 1243–1250. 10.4067/s0717-95022013000400016

[B22] Paoli A. Gentil P. Moro T. Marcolin G. Bianco A. (2017). Resistance training with single vs. multi-joint exercises at equal total load volume: Effects on body composition, cardiorespiratory fitness, and muscle strength. Front. Physiol. 8, 1105. 10.3389/fphys.2017.01105 29312007 PMC5744434

[B23] Rahimi R. (2006). Effect of moderate and high intensity weight training on the body composition of overweight men. Facta Univ. Phys. Educ. Sport 4 (2), 93–101.

[B24] Ross R. Soni S. Houle S. (2020). Negative energy balance induced by exercise or diet: Effects on visceral adipose tissue and liver fat. Nutrients 12 (4), 891. 10.3390/nu12040891 32218121 PMC7230996

[B25] Rutherford W. J. Diemer G. A. Scott E. D. (2011). Comparison of bioelectrical impedance and skinfolds with hydrodensitometry in the assessment of body composition in healthy young adults. ICHPER-SD J. Res. 6 (2), 56–60.

[B26] Saris W. H. M. Blair S. N. Van Baak M. A. Eaton S. B. Davies P. S. W. Di Pietro L. (2003). How much physical activity is enough to prevent unhealthy weight gain? Outcome of the IASO 1st stock conference and consensus statement. Obes. Rev. 4 (2), 101–114. 10.1046/j.1467-789x.2003.00101.x 12760445

[B27] Silva D. A. S. Petroski E. L. Pelegrini A. (2014). Effects of aerobic exercise on the body composition and lipid profile of overweight adolescents. Rev. Bras. Ciências do Esporte. 36, 295–309. 10.1590/s0101-32892014000200002

[B28] Špirtović O. Bajrić S. Hadžić R. (2021). Influence of programmed exercise on body composition indicators of recreational exercisers. Sport Sci. Heal Nauk. i Zdr. 11 (2).

[B29] Stojiljković S. Djordjević-Nikić M. Macura M. (2005). Influence of individual programmed exercises and nutrition on the body composition of recreational population. Abstr. book 10th Annu. Congr. Eur. Coll. Sport Sci., 173–182.

[B30] Vasold K. L. Parks A. C. Phelan D. M. L. Pontifex M. B. Pivarnik J. M. (2019). Reliability and validity of commercially available low-cost bioelectrical impedance analysis. Int. J. Sport Nutr. Exerc Metab. 29 (4), 406–410. 10.1123/ijsnem.2018-0283 30507268

[B31] Wargo A. R. Kurath G. (2012). Viral fitness: Definitions, measurement, and current insights. Curr. Opin. Virol. 2 (5), 538–545. 10.1016/j.coviro.2012.07.007 22986085 PMC7102723

[B32] Zouhal H. Ben Abderrahman A. Khodamoradi A. Saeidi A. Jayavel A. Hackney A. C. (2020). Effects of physical training on anthropometrics, physical and physiological capacities in individuals with obesity: A systematic review. Obes. Rev. 21 (9), e13039. 10.1111/obr.13039 32383553

