# Corrigendum: The effects of preventive aerobics mix on body composition in healthy adult women

**DOI:** 10.3389/fphys.2024.1451181

**Published:** 2024-07-29

**Authors:** Omer Špirtović, Ilma Čaprić, Mima Stanković, Dušan Đorđević, Benin Murić, Izet Kahrović, Rifat Mujanović, Raid Mekić, Borko Katanić, Igor Jelaska, Goran Sporiš

**Affiliations:** ^1^ Department of Biomedical Sciences, State University of Novi Pazar, Novi Pazar, Serbia; ^2^ Faculty of Sport and Physical Education, University of Nis, Nis, Serbia; ^3^ Montenegrin Sports Academy (MSA), Podgorica, Montenegro; ^4^ Faculty of Kinesiology, University of Split, Split, Croatia; ^5^ Faculty of Kinesiology, University of Zagreb, Zagreb, Croatia

**Keywords:** fitness, recreation, aerobics, women, body composition

In the published article, there was an error in **Affiliation 1**. Instead of “Faculty of Sport and Physical Education, University of Novi Pazar, Novi Pazar, Serbia”, it should be “Department of Biomedical Sciences, State University of Novi Pazar, Novi Pazar, Serbia.”

The authors apologize for this error and state that this does not change the scientific conclusions of the article in any way. The original article has been updated.

